# Depersonalization and a severe form of agoraphobia: A case report and review

**DOI:** 10.1192/j.eurpsy.2021.496

**Published:** 2021-08-13

**Authors:** C. Pedro Fernandes, B. Jorge, D. Freitas

**Affiliations:** 1 Psychiatry, Hospital de Braga, Braga, Portugal; 2 Serviço De Psiquiatria, Hospital de Braga, Braga, Portugal

**Keywords:** panic disorder, depersonalization, agoraphobia, case report

## Abstract

**Introduction:**

Depersonalization during panic attacks may be a feature of a subgroup of Panic disorder. Several studies suggest that such subgroup corresponds to a more clinically severe form of Panic Disorder, with earlier onset and a higher rate of comorbidity with other psychiatric disorders, such as obsessive-compulsive disorder and generalized anxiety disorder. It is also hypothesized that depersonalization during panic attacks may lead Panic disorder to evolve into Agoraphobia.

**Objectives:**

To present the case report of a patient with severe Agoraphobia, whose only symptom of Panic disorder was depersonalization.

**Methods:**

Description of a case report.

**Results:**

We describe the case of a 20-year-old woman who developed Agoraphobia after a single panic attack, during a physical education class, at the age of 13, with depersonalization symptoms only. After the attack, the patient stopped playing sports and engaging in any kind of activity in the absence of a trusted person. At the age of 20, the patient will only travel alone in the immediacies of her home, sometimes missing classes, because she cannot get a ride from trusted acquaintances. She justifies such avoidances with her fear of feeling depersonalized again. Over the course of her illness, she denied having experienced any other symptoms of a panic attack. She was treated with Paroxetine 40mg daily and cognitive behavioral therapy, having improved.

**Conclusions:**

We believe this case provides good insight into depersonalization in panic attacks, supporting the view that Panic disorder with depersonalization may be a distinct and more severe subgroup of Panic Disorder.

